# Structural Characterization and Anti-Tumor Activity of a Polysaccharide from *Laetiporus sulphureus* in A549 Cells

**DOI:** 10.3390/molecules30183706

**Published:** 2025-09-11

**Authors:** Yunhe Qu, Xing Yang, Dongxue Zhao, Pingping Zhang, Yue Mi, Jing Xu, Boya Zhao, Dongfang Shi

**Affiliations:** 1 Central Laboratory, Changchun Normal University, No. 677 North Changji Road, Changchun 130032, China; quyunhe@ccsfu.edu.cn (Y.Q.);; 2 School of Life Science, Changchun Normal University, No. 677 North Changji Road, Changchun 130032, China; zhaodongxx77@163.com

**Keywords:** *Laetiporus sulphureus*, β-glucan, structure, anti-tumor in A549 lung carcinoma, oxidative stress, apoptosis

## Abstract

While numerous bioactive polysaccharides have been identified from mushrooms, their mechanisms of action, particularly through the induction of oxidative stress in tumor cells, remain underexplored. This study isolates a novel polysaccharide, LSPS2, derived from *Laetiporus sulphureus*, followed by the elucidation of its distinctive structural features and specific antitumor activity in A549 lung carcinoma cells. LSPS2 was composed primarily of glucose (88.1%) and minor amounts of mannose (8.0%) and galactose (3.9%). Methylation and one-dimensional/two-dimensional nuclear magnetic resonance (1D/2D NMR) analysis results indicated that LSPS2 was composed of (1→3)-linked-D-β-glucopyran residues and (1→4)-linked-D-α-glucopyran residues as the main chain. The side chains were connected to O-6 and O-3 of glucopyranose (Glc*p*) residues with terminal Glc*p*. It differs from previous reports on *L. sulphureus* polysaccharides. Functionally, LSPS2 markedly suppressed A549 cell viability in a manner that depended on both exposure duration and concentration. LSPS2 upregulated malondialdehyde (MDA) and downregulated reduced glutathione (GSH), demonstrating that LSPS2 induces oxidative stress in A549 cells. The results of superoxide dismutase (SOD) activity assays further indicated that LSPS2 downregulates SOD activity, which may be the mechanism by which LSPS2 induces oxidative stress and, consequently, apoptosis in A549 cells. This targeted downregulation of a key antioxidant enzyme highlights a potential pathway for polysaccharide-induced tumor cell death. Our findings not only identify LSPS2 as a structurally distinct biopolymer but also elucidate its mode of action, underscoring its prospective application in tumor therapy and functional foods, warranting further investigation.

## 1. Introduction

The global edible mushroom industry is growing rapidly and has significant potential within the food and pharmaceutical sectors. Edible mushrooms are rich in nutrients such as vitamins, minerals, and essential amino acids [1], as well as bioactive components including polysaccharides [2], polyphenols, proteins [3], and proteoglycans [4,5]. Polysaccharides are particularly important among these components [6]. Within fungal organisms, polysaccharides primarily serve fundamental structural and energy storage roles. When isolated and administered externally to humans or animal models, these same polysaccharides can exhibit indirect biological effects, such as immunomodulation, or direct antitumor activities, which are not their primary function in the native organism.

**Figure 1:**
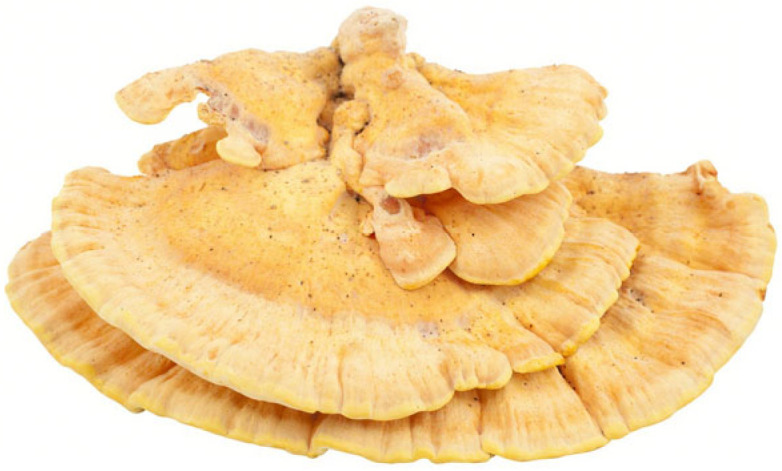
Image of *Laetiporus sulphureus* (*L. sulphureus*).

*Laetiporus sulphureus* (*L. sulphureus*) (Figure 1), which is commonly referred to as yellow linzhi or “chicken of the woods”, serves as both food and medicine, taxonomically placed within the genus *Laetiporus* in the family Formitopsidaceae and the order Polyporales [7]. The structure is characterized by a shelf-like configuration, with a vivid yellow or orange hue [8]. The consumption of *L. sulphureus*, along with its utilization as a medicinal agent, has a long-standing tradition in Asia and Europe. Research has demonstrated that this species contains a variety of bioactive substances, including polysaccharides, lectins [9], polyene lactoferrins, triterpenoids [10], and volatile metabolites. *L. sulphureus* is notably abundant in glucans. Wiater et al. extracted an α-1,3-linked glucan from *L. sulphureus* carpophores. The derived oligosaccharides exhibited selective enhancement of beneficial microbial growth [11]. Lu et al. successfully extracted sulfated galactoglucan from *L. sulphureus*, designated SPS, using papain-assisted hydrolysis. SPS exhibits anti-inflammatory and anti-cancer properties by suppressing lipopolysaccharide-induced TNF-α secretion in RAW264.7 macrophages [12]. Several fractions of the sulfated *L. sulphureus* polysaccharides prepared using the papain-assisted hydrolysis method display diverse capacities to suppress the proliferation of MDA-MB-231 human breast carcinoma cells, based on the research findings of Jen et al. [13]. However, detailed structural characterization studies of purified polysaccharide components derived from *L. sulphureus* are relatively scarce. Furthermore, research on their precise antitumor activity mechanisms, particularly those directly related to the induction of tumor cell apoptosis, is even more limited.

The global prevalence of cancer has led to a resurgence of interest in low-toxicity, safe natural treatments, which have occurred as a noticeable area. Evidence indicates that fungal polysaccharides suppress tumorigenesis via multiple pathways, including inducing apoptosis [14], curbing proliferation [15] and angiogenesis, while also modulating the host immune response [16]. Certain natural polysaccharides from fungi exhibit potent antitumor activity with minimal toxic side effects in cellular and animal model, offering a promising therapeutic avenue [17]. For instance, *Poria cocos* polysaccharide [18], *Grifola frondosa* polysaccharide [19], *Astragalus* polysaccharides, among others, have been developed as clinical drugs. Apoptosis represents a tightly regulated cell death that is central to many anti-cancer treatments, including natural products [20]. This process is orchestrated through multiple signaling pathways and molecular mechanisms. The redox system is crucial in this process by adjusting the balance of redox reactions to influence cellular survival and death [21]. This system comprises various molecules and enzymes, including superoxide dismutase (SOD), glutathione (GSH) and glutathione peroxidase (GPx), which work together to maintain the balance of redox reactions within cells [22]. SOD may influence cell survival or death by regulating oxidative stress levels during apoptosis [23]. Glutathione (GSH) is one of the most important antioxidants in cells and maintains redox balance through the dynamic equilibrium between its reduced (GSH) and oxidized (GSSG) forms. GSH plays a pivotal role in apoptosis; decreased levels or an altered oxidative state can activate apoptotic signaling pathways. For instance, the depletion or oxidation of GSH can initiate apoptosis, potentially via activation of the mitochondrial or death receptor pathways. Increased GSH oxidation (GSSG) can also trigger apoptosis, particularly under oxidative stress conditions [24]. Lipid peroxidation is an important manifestation of oxidative stress, and its end products, such as malondialdehyde (MDA), are commonly used as markers of oxidative damage. Not only does the accumulation of MDA disrupt lipid membrane structure and cause organelle dysfunction, it may also promote apoptosis by activating the mitochondrial apoptosis pathway. Therefore, elevated MDA levels are typically closely associated with cellular damage and apoptosis [25]. Edible mushroom polysaccharides are capable of activating the human immune system by stimulating a variety of immune cells, producing cytokines, and other mechanisms to inhibit tumor cell growth or directly kill tumor cells. The substance exerts its effects by regulating the redox balance within cells [26,27]. Apoptosis of cancer cells is closely related to their oxidative stress, as reported in several studies [28,29,30,31]. Polysaccharides can trigger reactive oxygen species (ROS) over-generation that surpasses the antioxidant defenses of tumor cells, provoking oxidative stress. Persistent oxidative stress is known to engage the mitochondrial, death receptor or endoplasmic reticulum-mediated routes to programmed cell death. This activation, in turn, can ultimately trigger a cascade of reactions involving the enzyme caspase [32], which in turn results in the process of cell apoptosis [33,34].

In the present study, a previously uncharacterized polysaccharide was isolated and purified from *L. sulphureus*. In addition, its structural characteristics were analyzed, and its antitumor activity was evaluated. Our experiments provide important data and theoretical support for the development and application of polysaccharides derived from *L. sulphureus*.

## 2. Results and Analysis

### 2.1. Obtained L. Sulphureus Polysaccharide

**Figure 2:**
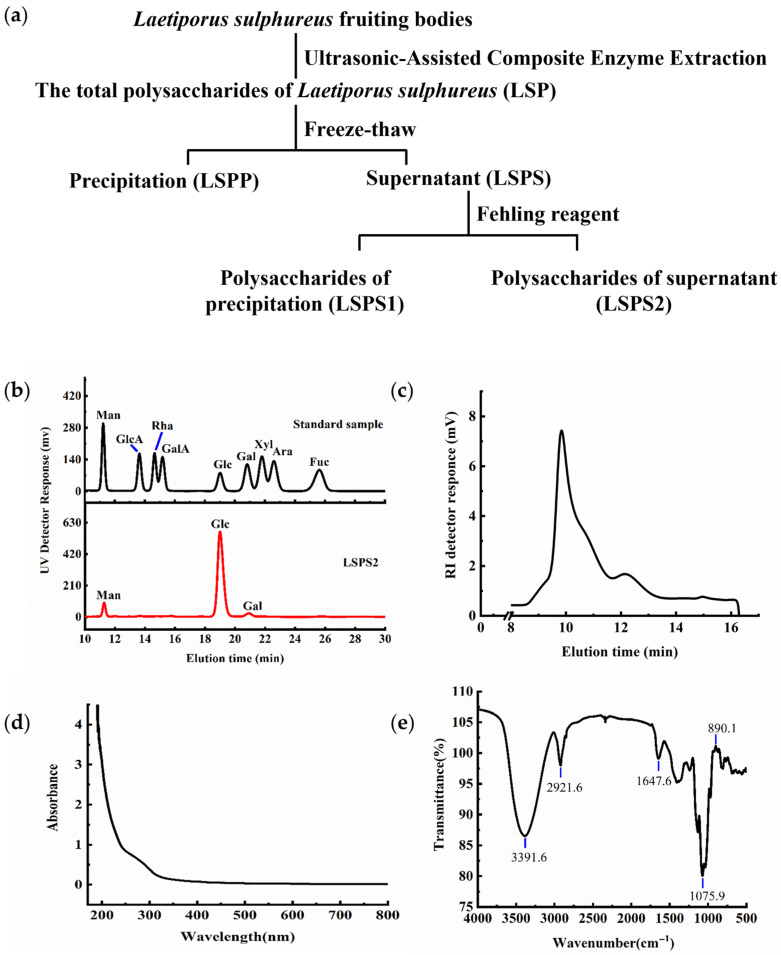
Flowchart of *L. sulphureus* polysaccharide extraction and determination of its physicochemical properties. (**a**) Flowchart of LSPS2 extraction. (**b**) The monosaccharide composition of LSPS2. (**c**) The molecular weight distribution of LSPS2. (**d**) Ultraviolet–visible (UV-vis) Spectroscopy of LSPS2. (**e**) Infrared Spectroscopy (IR) of LSPS2. RI, refractive index.

*L. sulphureus* polysaccharide (LSPS2) was achieved from *L. sulphureus* via ultrasonic-assisted composite enzyme extraction and separated using the Fehling’s reagent method as described in our previous report [35]. LSPS2 was successfully isolated from LSP through a purification process, achieving a yield of 33.2%, as determined through gravimetric analysis (Figure 2a). Monosaccharide composition analysis revealed that LSPS2 is a heteropolysaccharide consisting of three constituent sugars: Glc, mannose (Man), and galactose (Gal). Quantitative analysis showed their molar ratio to be 88.2:8.9:3.0, respectively (Figure 2b), indicating that Glc is the predominant monosaccharide unit. Our molecular weight distribution analysis results showed that LSPS2 exhibits a polydisperse molecular weight distribution (Figure 2c). The broad elution profile suggests the presence of multiple polysaccharide populations with varying degrees of polymerization, resulting in a non-homogeneous molecular weight distribution. This heterogeneity is a characteristic feature of biologically derived polysaccharides and may contribute to the functional properties of the compound.

### 2.2. UV-Vis and FT-IR Analysis

The UV-Vis spectroscopic analysis of LSPS2, as depicted in Figure 2d, revealed a notable absence of characteristic absorption peaks at 260 nm and 280 nm. This spectral feature indicates the elimination of nucleic acid (260 nm) and protein (280 nm) contaminants, thereby confirming the exceptional purity of LSPS2 [36]. The Fourier Transform Infrared (FT-IR) spectrum (Figure 2e) exhibited distinctive absorption bands that are diagnostic of a carbohydrate’s molecular structure. A broad and intense absorption band centered at 3391.6 cm^−1^ was observed, which is characteristic of the O-H stretching vibration typically associated with hydroxyl groups in polysaccharides. The absorption peak at 2921.6 cm^−1^ corresponds to the C-H stretching vibrations of methylene groups. The spectral feature at 1647.6 cm^−1^ can be attributed to the bending vibration mode of C-H bonds. Furthermore, the strong absorption band at 1075.9 cm^−1^ is indicative of C-O-C glycosidic bond stretching vibrations, which are fundamental to polysaccharide backbone structures. Of particular significance is the absorption peak at 890.1 cm^−1^, which serves as a definitive spectroscopic marker for the β-configuration of the glycosidic linkages in the polysaccharide structure [37,38].

### 2.3. Methylation Analysis

**Figure 3:**
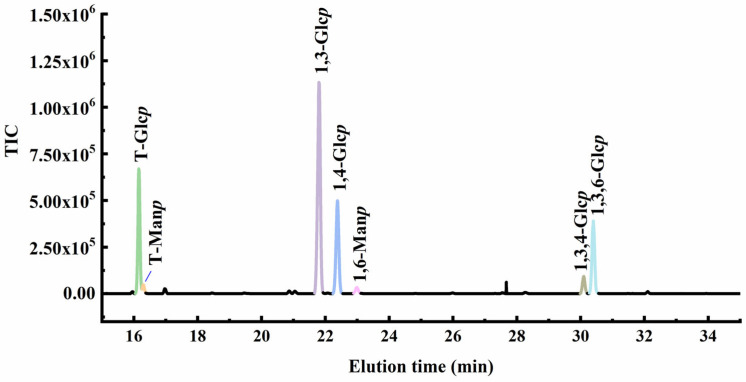
Methylation analysis of *L. sulphureus* polysaccharide (LSPS2) as determined by gas chromatography–mass (GC-MS).

To elucidate the glycosyl linkage in LSPS2, comprehensive methylation analysis was performed following established protocols. The polysaccharide was subjected to sequential methylation, acid hydrolysis, reduction, and peracetylation to generate partially methylated alditol acetate (PMAA) derivatives for GC-MS analysis (Figure 3 and see Supplementary Materials). Considering our methylation results and monosaccharide composition, we concluded that 1,3,5-Tri-O-acetyl-1-hydrogen-2,4,6-tri-O-methyl-D-glucitol (indicating 3-linked Glc*p* residues), 1,4,5-Tri-O-acetyl-1-hydrogen-2,3,6-tri-O-methyl-D-glucitol (indicating 4-linked Glc*p* residues), 1,5-Di-O-acetyl-1-hydrogen-2,3,4,6-tetra-O-methyl-D-glucitol (terminal Glc*p*), and 1,3,5,6-Tetra-O-acetyl-1-hydrogen-2,4-di-O-methyl-D-glucitol (3,6-linked Glc*p* residues) were the primary fragments in LSPS2 together with minor amounts of 1,3,4,5-Tetra-O-acetyl-1-hydrogen-2,6-di-O-methyl-D-glucitol (3,4-linked Glc*p*), 1,5,6-Tri-O-acetyl-1-hydrogen-2,3,4-tri-O-methyl-D-mannitol (6-linked Man*p*), and 1,5-Di-O-acetyl-1-hydrogen-2,3,4,6-tetra-O-methyl-D-mannitol (terminal Man*p*). From the above results, we inferred that the main chain of LSPS2 consisted of (1→3)-linked-D-Glc*p* and (1→4)-linked-D-Glc*p* residues. The side chains were connected to O-6 and O-3 with terminal Glc*p*, terminal Man*p*, and (1→6)-linked-D-Man*p* (Table 1).

### 2.4. NMR Analysis

**Figure 4:**
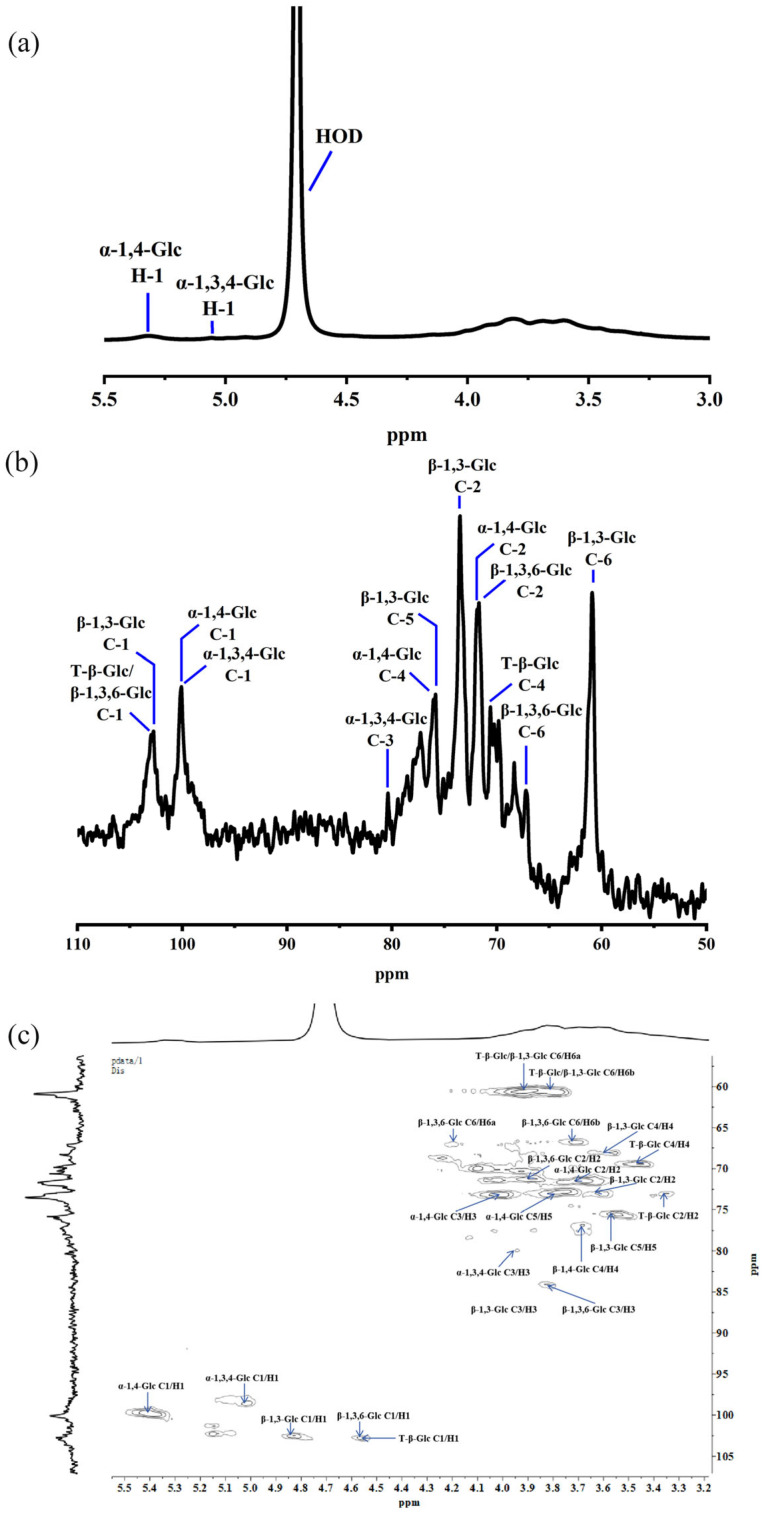
Determination of the structure of LSPS2. (**a**) ^1^H NMR analysis. (**b**) ^13^C NMR analysis. (**c**) Heteronuclear Single Quantum Correlation (HSQC) analysis.

The structure of the heteropolysaccharide LSPS2 was characterized using NMR spectroscopy, including ^1^H NMR, ^13^C NMR, and 2D HSQC experiments (Figure 4). In the anomeric range (4.5–5.5 ppm) of the ^1^H NMR spectrum, three distinct proton peaks were discovered at 5.39, 5.02, and 4.70 ppm. In addition, the 3.0–4.5 ppm region exhibited overlapping signals attributed to the ring protons (H2–H6) of the sugar residues, consistent with typical polysaccharide spectra. The peaks at 102.93 ppm, 102.70 ppm, and 102.48 ppm in the anomeric carbon region (90–110 ppm) of the ^13^C NMR spectrum revealed key structural information and indicated that some residues were β-configured [39,40]. Peaks at 100.01 ppm and 98.66 ppm suggested that other residues were α-configured [41,42]. Cross-peak signals at 5.39/100.01 ppm, 5.02/98.66 ppm, 4.83/100.01 ppm, 4.56/102.70 ppm, and 4.56/102.93 ppm in HSQC were attributed to δH-1/δC-1 of 4→)-α-D-Glc*p*-(1→, 3,4→)-α-D-Glc*p*-(1→, 3→)-β-D-Glc*p*-(1→, 3,6→)-β-D-Glc*p*-(1→ and β-D-Glc*p*-(1→, respectively. The other δH/δC signals of these five sugar residues and their chemical shifts are exhibited in Table 2 [43,44]. In combination with methylation analysis, the composition of LSPS2 is most likely (1→3)-linked β-D-glucan and (1→4)-linked α-D-glucan, with a backbone branched at the O-6 and O-3 of Glc*p* by β-Glc*p* side chains.

### 2.5. The Structural Differences Between LSPS1 and LSPS2

Our previous research prepared a *L. sulphureus* polysaccharide named LSPS1 [34]. LSPS1, included as an intra-species benchmark, contrasts with LSPS2 in polysaccharide architecture, underscoring the latter’s structural novelty. LSPS1 is a heteropolysaccharide primarily composed of Gal, Man, fucose, and Glc in a ratio of 10.06:5.22:3.13:1.00. Its molecular weight represents approximately 17.3 kDa. And the major chain structure is 1,6-linked galactopyranose (→6)-Gal*p*-(1→) residues, bearing single t-Man*p*, t-Fuc*p,* and t-Glc*p* substituents solely at O-2 position. In contrast, LSPS2 is a heteropolysaccharide dominated by Glc (88.1%), containing only minor amounts of Man (8.0%) and Gal (3.9%). Comprehensive analysis via methylation and 1D/2D NMR spectroscopy established that LSPS2 possesses a Glc-based backbone featuring both (1→3)-linked-β-D-glucopyranose and (1→4)-linked-α-D-glucopyranose residues. This backbone is substituted at the O-6 and O-3 positions of glucopyranose residues, terminating exclusively in terminal glucopyranose (t-Glc*p*) units. Consequently, LSPS1 represents a branched galactan with heterogeneous O-2-linked termini, while LSPS2 is characterized as a mixed-linkage (α/β) glucan with branching occurring at multiple positions (O-3 and O-6).

### 2.6. LSPS2 Decreased A549 Cell Viability

**Figure 5:**
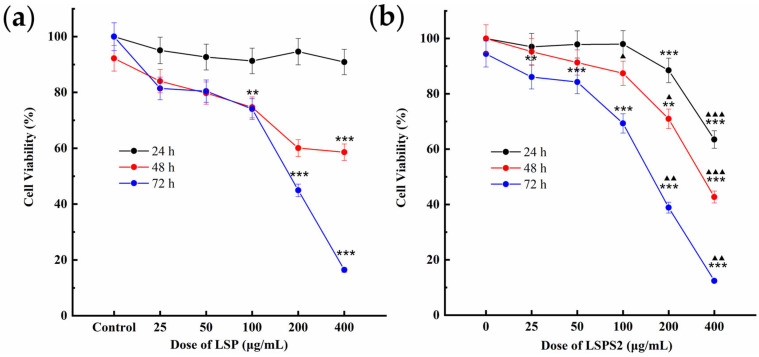
Impact of (**a**) LSP and (**b**) LSPS2 treatment on A549 cell viability. Data were represented as the mean ± SD (*n* = 3). Asterisks denote significant differences versus the Con group (** *p* < 0.01, *** *p* < 0.001) and triangles indicate significant differences relative to LSP at identical concentration and duration (^▲^ *p* < 0.05, ^▲▲^ *p* < 0.01, ^▲▲▲^ *p* < 0.001).

To determine the effect of LSP (crude polysaccharide) and LSPS2 (purified polysaccharide) treatment on A549 cell viability, an in vitro cell viability assay was conducted. A549 cells were cultured in the presence of increasing concentrations (0, 25, 50, 100, 200, or 400 μg/mL) of LSP or LSPS2 for 24, 48, and 72 h. Cell viability was quantitatively assessed via the Cell Counting Kit-8 (CCK-8) assay, which measures cellular metabolic activity as a surrogate for viability. As illustrated in Figure 5, both LSP and LSPS2 markedly suppressed A549 viability in proportion to concentration and exposure duration relative to untreated controls. At 24 h and 48 h, LSPS2 at 200 and 400 µg/mL showed a significant inhibitory effect on A549 cell viability. At 72 h, significant inhibition was achieved by LSP at 100, 200, and 400 µg/mL, and LSPS2 over an extended range from 25 to 400 µg/mL. Crucially, LSPS2 demonstrated significantly more pronounced effects in comparison to LSP at equivalent doses (200 and 400 µg/mL) and times (48 and 72 h). This enhanced inhibitory effect of LSPS2 suggests that the purification process may have concentrated bioactive components responsible for the observed antitumor activity.

### 2.7. Influence of LSPS2 Treatment on the Oxidative Stress of A549 Cells

**Figure 6:**
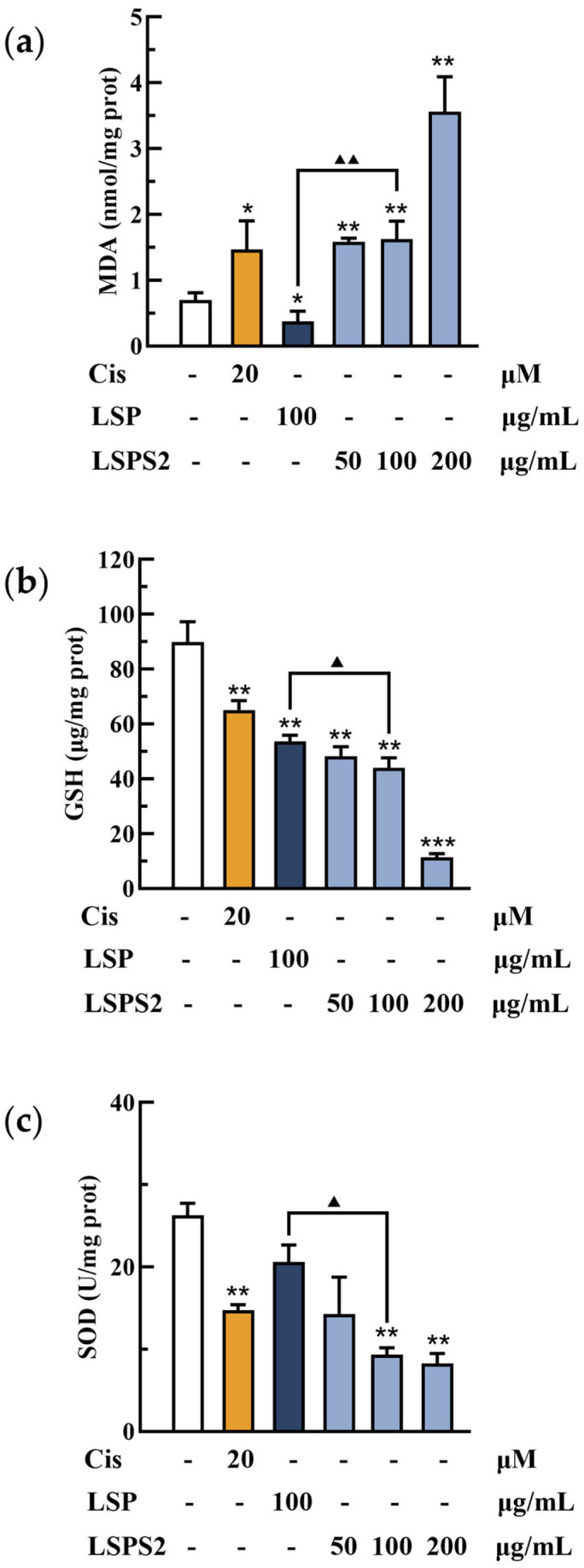
Influence of LSPS2 treatment on oxidative stress of A549 cells. (**a**) The content of malondialdehyde (MDA). (**b**) The content of glutathione (GSH). (**c**) The content of superoxide dismutase (SOD). Con, blank control group. Cis, positive control group. Data were shown as the mean ± SD (*n* = 3, * *p* < 0.05, ** *p* < 0.01, *** *p* < 0.001 compared with the Con group, ^▲^ *p* < 0.05, ^▲▲^ *p* < 0.01, compared between two groups). All experiments were performed in triplicate.

**Figure 7:**
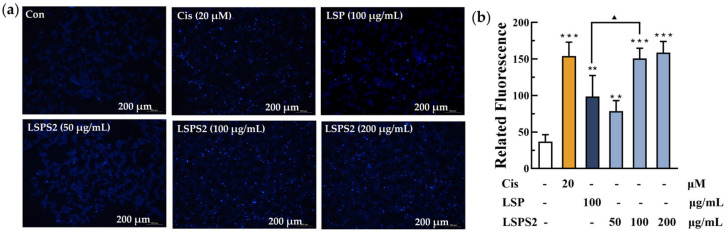
(**a**) The fluorescence images and (**b**) the statistical analysis of LSPS2-induced cell apoptosis. (Each bar represents the mean ± SD of four groups); ** *p* < 0.01, *** *p* < 0.001 relative to the group of Con; ^▲^ *p* < 0.05 assessed between LSP of 100 μg mL^−1^ and LSPS2 of the same concentration.

Oxidative stress serves as a pivotal mechanistic pathway in triggering the apoptosis of cancer cells. To evaluate the pro-oxidative effects of LSP and LSPS2 compounds in A549 adenocarcinoma cells, key biomarkers of oxidative stress—MDA and reduced GSH—were quantitatively analyzed. Our experimental data demonstrated that LSPS2 treatment elicited a significant dose-dependent increase in MDA content, coupled with marked depletion of intracellular GSH levels (Figure 6a,b). These findings collectively indicate substantial oxidative stress induction in A549 cells following compound exposure. The results indicated that 100 μg/mL of LSP2 significantly elevated MDA levels while reducing GSH levels in A549 cells compared to LSP, suggesting a stronger capacity to induce oxidative damage. SOD, a critical metalloenzyme in the cellular antioxidant defense system, plays a crucial role in keeping redox homeostasis by catalyzing the dismutation of superoxide radicals (O_2_•^−^) into hydrogen peroxide and molecular oxygen. This enzymatic activity prevents lipid peroxidation cascades and preserves GSH from oxidative depletion. Intriguingly, while LSPS2 at the low concentration did not significantly alter SOD activity, treatment with higher concentrations (200 µg/mL) resulted in a marked downregulation of SOD activity compared to the control group (Figure 6c). The observed SOD inhibition likely contributes to exacerbated lipid peroxidation (evidenced by elevated MDA levels) and compromised antioxidant capacity (reflected by GSH depletion). This tripartite effect establishes a pro-oxidant cellular environment that may drive A549 cells toward apoptotic cell death. As demonstrated in Figure 7, both LSP at 100 μg/mL and LSPS2 (50–200 μg/mL) have the capacity to induce cell apoptosis. At an equivalent concentration of 100 μg/mL, LSPS2 demonstrates a more pronounced apoptotic effect in comparison to LSP. The apoptotic effect of LSPS2 is concentration-dependent. The above data suggest a model wherein LSPS2 preferentially targets the SOD-mediated antioxidant system to amplify oxidative stress beyond cellular tolerance thresholds, thereby inducing regulated cell death in malignant cells. The differential response between LSP and LSPS2 at equivalent doses suggests structure–activity relationships that warrant future structure–function analyses.

## 3. Discussion

In the present, we isolated and prepared a heterogeneous polysaccharide, designated LSPS2, from *L. sulphureus*, which exhibited a wide molecular weight range. Polysaccharides with low molecular weight have been considered to be more readily absorbed and utilized by the body; however, they may possess an insufficient degree of structural complexity to effectively activate the immune system. High-molecular-weight polysaccharides have been shown to possess immune-stimulating activity; however, this activity may be impaired due to low solubility or difficulty in penetrating cell membranes [45,46,47]. The heterogeneous separation of LSPS2 preserves the functional integrity of the natural extract [48].

The functional effects of polysaccharides are closely related to their structural specificity. The results presented in this study demonstrate that the polysaccharide LSPS2, purified from *L. sulphureus*, exhibits a Glc–Man–Gal ratio of approximately 88.2:8.9:3. The polysaccharide in question, which is primarily composed of (1→3)-β-D-glucan and (1→4)-α-D-glucan residues, has been shown to enhance oxidative stress by inhibiting SOD activity in A549 cells. This inhibition is manifested by a reduction in GSH and a concomitant rise in MDA content, thereby inducing apoptosis. In contrast, for the polysaccharides PSI (Arabinose–Man–Glc–Gal ≈ 1:6.2:6.3:67.2) and PSII (Xylose–Glc–Gal ≈ 1:83.9:4.2) from *Pleurotus citrinopileatus*, an opposing regulatory effect is exhibited: PSI and PSII from *Pleurotus citrinopileatus* markedly enhance HepG2 antioxidant capacity and survival, restore ALT/AST to baseline, and lower triglyceride (TG) levels. This finding indicates that both polysaccharides could serve as promising agents against fatty liver disease [49]. The regulation of SOD activity is bidirectional: LSPS2 induces pro-apoptotic oxidative stress by inhibiting SOD, whereas PSI/PSII exerts cell protective and liver injury repair functions by enhancing SOD activity.

Polysaccharide isolation from edible fungi typically combines heat-assisted aqueous, acidic, or alkaline extraction with ultrasonic [50] or enzyme assistance [51], followed by chromatographic purification. In this study, LSPS2 was achieved through the utilization of hot water extraction and Fehling’s reagent method. The final yield of LSPS2 was 33.2% relative to total polysaccharides (Figure 2a), which, to a certain extent, retained the integrity of the glucan, which is generally associated with effective antitumor activity. The findings of other research groups reported that the yield for *L. sulphureus* sulfated polysaccharides can be enhanced through the application of hot water extraction, papain-assisted extraction, and dialysis purification, with a reported yield of 9.75%.

In this study, we investigated the antitumor activity of LSPS2 in vitro and its mechanism of action. The results demonstrate that LSPS2 triggers oxidative stress-mediated apoptosis in A549 lung cancer cells, characterized by increased MDA content, decreased GSH levels, and reduced SOD activity. This mechanism contrasts with but complements the anti-proliferative actions of sulfur-rich polysaccharides (F2/SPS) from *L. sulphureus* reported in earlier studies on MDA-MB-231 breast cancer cells [13,52,53]. In those works, F2/SPS—enriched in sulfate and protein—suppressed proliferation by halting cells in G0/G1 via CDK4/cyclin D1 downregulation and p21 upregulation, rather than directly inducing apoptosis at early timepoints. The divergent mechanisms—oxidative stress/apoptosis in A549 versus EGFR-pathway-mediated cell cycle arrest in MDA-MB-231—likely arise from structural differences in the polysaccharides, particularly sulfate content and monosaccharide composition, which collectively influence receptor targeting and bioactivity. Importantly, all studies underscore the broad therapeutic potential of *L. sulphureus* polysaccharides. The induction of apoptosis and the regulation of oxidative stress are two common mechanisms through which naturally sourced polysaccharides exert their antitumor effects. Enzymatically hydrolyzed polysaccharides derived from *Ganoderma lucidum* have been shown to induce apoptosis in human colon cancer cells (HCT-116). This process is achieved by upregulating the expression of BCL-2-associated X protein (Bax) and phospho-extracellular signal-regulated kinase (P-ERK). It has been demonstrated that cleaved caspase-3 can induce apoptosis in human colon cancer cells (HCT-116) through a number of mechanisms. Firstly, it has been shown to downregulate the expression of B-cell lymphoma-2 (Bcl-2), phosphorylated serine/threonine kinase 1 (p-Akt1), and cyclooxygenase-2 (COX-2) [54]. The investigation revealed that *Astragalus* polysaccharides exhibited the capacity to impede the proliferation of lung cancer cells (A459 cells) and concomitantly acted synergistically with metformin against A549 cells [55].

In summary, in this study, we successfully isolated a heterogeneous polysaccharide from *L. sulphureus*. This polysaccharide has been demonstrated to possess antitumor activity. However, the study’s in vivo validation is lacking. While these findings highlight the potential of LSPS2 as an anti-cancer agent targeting oxidative stress, further in vivo validation remains essential to corroborate efficacy and explore possible synergism with conventional therapies. Future work should also address the purification, scalability, and detailed signaling pathways involved. As this study represents only an initial exploration of the antitumor potential of LSP2, we acknowledge the current limitations in evaluating tumor malignancy comprehensively. Consequently, the findings of this study are considered preliminary, and further research in vivo is imperative to substantiate any prospective therapeutic benefits. A comprehensive analysis revealed that the molecular weight, monosaccharide composition, and molecular structure of LSPS2 are pivotal in its biological activity, achieving antitumor effects by regulating oxidative stress-induced apoptosis.

## 4. Materials and Methods

### 4.1. Materials

*L. Sulphureus* fruiting bodies were obtained at a local supplier in Changchun, China. They were authenticated through rDNA-ITS sequencing analysis. Lung cancer A549 cells and DMEM complete culture medium (PM150210-500) were supplied by Procell Life Technology Co., Ltd. (Procell, Wuhan, China). A CCK8 kit was procured from Biotoda Technology Co., Ltd. (Biotoda, Beijing, China). Commercialized reagent kits for oxidative stress markers were purchased from Beijing Solarbio Science & Technology Co., Ltd. (Solarbio, Beijing, China). All remaining reagents were analytical grade and sourced from standard vendors.

### 4.2. Extraction and Purification of L. sulphureus Polysaccharide

*L. sulphureus* polysaccharide (LSPS2) was prepared using a previously reported method [35]. The ultrasound-assisted complex enzyme method was used to extract polysaccharides from *L. sulphureus*. After 50 g of dried substrates were pulverized, dispersed in distilled water (1:30 *w*/*v*), supplemented with 3% (*w*/*w*) of an acid-protease/laccase/cellulase mixture (1:1:1), and ultrasonicated at 50 °C for 30 min; thereafter, enzymatic extraction was continued for 80 min. Next, 65% ethanol was used for final concentration alcohol precipitation and centrifuged, and the precipitate was lyophilized to obtain the polysaccharide referred to as LSP. LSP was prepared as a 10% aqueous solution, frozen at −20 °C for 24 h, and then thawed at 4 °C and centrifuged to obtain the freeze–thawed supernatant (LSPS). Following successive freeze–thaw cycles until precipitation ceased, LSPS (50 mg) was dispersed in Fehling’s Reagent B (2.5 mL), briefly vortex-mixed, then combined with an equal volume of Fehling’s Reagent A. The mixture was magnetically stirred at room temperature for 6 h. The supernatant was collected via centrifugation and neutralized with 10% acetic acid, and then dialyzed. The dialysate was added to cationic exchange resin with stirring for 1 h and then lyophilized to obtain LSPS2.

### 4.3. Analysis of the Composition for Monosaccharide

The composition of monosaccharide was detected using the protocol reported by Li et al. [56]. A total of 1 mg of LSPS2 was first methanolized in 1 mL of anhydrous methanol—2 M HCl. The reaction was carried out for 16 h at 80 °C, and thereafter hydrolyzed with 2 M of trifluoroacetic acid (120 °C, 1 h). Following the derivation by 1-phenyl-3-methyl-5-pyrazolone, the derivatives were analyzed by a high-performance liquid chromatography (HPLC) system. The following conditions were applied for HPLC analysis of monosaccharide composition: Instrument: Shimadzu LC-10 AT VP HPLC with UV (Kyoto, Japan), detector column: COSMOSIL 5C_18_-PAQ 4.6 ID × 250 mm (NACALATESOUE, INC., Kyoto, Japan), detection temperature at 35 °C, and wavelength at 245 nm. The eluent consists of PBS with a pH of 7.0 and a concentration of 0.1 M, and acetonitrile, with a volume ratio of 80.8:19.2. The flow rate is 1.0 mL/min.

### 4.4. The Distribution of Molecular Weight

The distribution of molecular weight was detected using high-performance gel permeation chromatography (HPGPC) on a column of TSK-gel G-3000PW_XL_ (7.8 × 300 mm, TOSOH, Tokyo, Japan) linked to a Shimadzu HPLC system [56]. Dextrans of 50, 25, 12, 5, and 1 kDa served as external standards, yielding the calibration equation Y = −0.4327x + 9.5898 (R^2^ = 0.9989).

### 4.5. UV-Vis Spectroscopy

An accurately weighed LSPS2 was added to distilled water and dissolved to yield a 1 mg/mL solution. The ultraviolet–visible absorption profile was recorded between 200 and 900 nm using a Shimadzu UV-2700 spectrophotometer (Japan).

### 4.6. Infrared Spectroscopy

Next, 1~2 mg of the sample was homogenized with an appropriate amount of KBr (~200 mg), thoroughly ground, and compacted into a pellet. IR spectra were recorded using a Spectrum Two Infrared spectrometer (PerkinElmer, Waltham, MA, USA) in the range of 4000–400 cm^−1^. Methylated samples were dissolved in dichloromethane, and 50 μL of the solution was added to a potassium bromide plate, dried, and analyzed using IR.

### 4.7. Analysis of Methylation

The analysis of methylation was performed based on Needs and Selvendran [57]. Briefly, 5 mg of LSPS2 was weighed, then 0.5 mL of DMSO was added. A total of 0.5 mL of sodium hydroxide–DMSO suspension was added, and thereafter methylated with 1 mL of iodomethane. The product was extracted with CH_2_Cl_2,_ dried, and judged completely methylated by the disappearance of the O–H stretch (3200–3400 cm^−1^) in Fourier transform Infrared Spectroscopy. The per-O-methylated polysaccharide was hydrolyzed successively with 1 mL 85% formic acid (100 °C, 4 h) and 1 mL 2 M trifluoroacetic acid (100 °C, 6 h). Following NaBH_4_ reduction and acetylation treatment, partially methylated sugars were detected using gas chromatography-mass spectrometry (GC-MS). The GC-MS model was the Agilent 7890B-5977B from the Santa Clara, CA, USA, and the column used was an HP-5 ms capillary column (30 m × 0.32 mm × 0.25 mm). The oven temperature varied from 120 °C (1 min) to 210 °C (2 min) at 3 °C/min, then to 260 °C (4 min) at 10 °C/min. Injector and detector were held at a temperature of 300 °C; carrier gas: helium and the MS scan from 50 to 500 *m*/*z*.

### 4.8. NMR Analysis

^1^H NMR, ^13^C NMR, and Heteronuclear Single Quantum Correlation (HSQC) NMR spectral analysis were performed on an AVANCEII 500 MHz (^1^H) (Bruker, Fällanden, Switzerland) spectrometer at 25 °C. A total of 20 mg of LSPS2 was added to 0.5 mL of D_2_O and dissolved. It was clarified by centrifuging at 12,000 rpm for 5 min, and the liquid layer floating on the surface was subjected to NMR analysis using Bruker TopSpin software (version 4.1.4).

### 4.9. Cell Culture

A549 cells (ATCC CCL-185) were grown at 37 °C in a humidified atmosphere containing 5% CO_2_. The basal medium was Dulbecco’s Modified Eagle’s Medium (DMEM), supplemented with 10% heat-inactivated fetal bovine serum (FBS), 2 mM L-glutamine, and 1% penicillin–streptomycin antibiotic solution (*v*/*v*). The complete growth medium was replaced every two to three days, and cells were sub-cultured at 80–90% confluence after brief exposure to 0.25% trypsin EDTA. Culture vessels were kept in a CO_2_ incubator that maintained a pH of 7.2–7.4 through bicarbonate buffering. The vessels were regularly monitored for Mycoplasma contamination.

### 4.10. Cell Viability Assay

Logarithmic-phase A549 cells were seeded at 1 × 10^3^ cells per well in 96-well plates (100 μL complete medium). After attachment, cultures were exposed to graded doses of crude (25, 50, 100, 200, and 400 μg/mL) and purified polysaccharide for 24, 48 or and 72 h. Next, cell viability was assessed by adding 10 μL CCK-8 reagent, incubating for 2 h, and measuring absorbance at 450 nm with a microplate reader of the model Spark produced by TECAN (Männedorf, Switzerland). Data from three biologically independent experiments (each experiment was repeated three times) are presented as mean ± SD relative to vehicle control (defined as 100%).

### 4.11. Determination of Cellular MDA, GSH, and SOD

Logarithmic-phase A549 human lung adenocarcinoma epithelial cells were harvested and prepared as a single-cell suspension using complete culture medium (F12 Ham’s medium containing fetal bovine serum (10%) and 2 mM L-glutamine). The suspensions of cells were seeded into 6-well culture plates at a density of 1 × 10^5^ cells per well, and incubated overnight (37 °C, 5% CO_2_, humidified atmosphere) to ensure attachment. Upon adherence, the medium was exchanged for fresh DMEM supplemented with crude polysaccharide (100 μg/mL) or graded doses (50, 100, and 200 μg/mL) of purified polysaccharide. The cells were maintained under these treatment conditions for 72 h with regular microscopic observation to monitor cell morphology and confluence. Following treatment, the cells were collected via gentle centrifugation at 1000 rpm (approximately 200× *g*) for 3 min at 4 °C. After two washes with ice-cold phosphate-buffered saline (PBS, pH 7.4), the cell pellet was resuspended in 50 μL of the same buffer. Cell lysis was achieved through three consecutive freeze–thaw cycles in a water bath alternating between −80 °C (15 min) and 37 °C (5 min) with gentle vortexing between cycles. Total cellular proteins were extracted from the lysate via centrifugation at 12,000× *g* (15 min, 4 °C) to remove cellular debris. The protein concentration in the supernatant was determined using the bicinchoninic acid (BCA) assay according to the manufacturer’s protocol, with bovine serum albumin used as the standard. The levels of MDA, reduced GSH, and SOD activity were measured according to the instructions provided in commercial test kits. Cisplatin was used as a positive control to validate the experimental model for inducing oxidative stress, given its well-documented ability to trigger robust oxidative damage and apoptosis in tumor cells. Consistent with its known mechanism, treatment with cisplatin resulted in a significant increase in lipid peroxidation (MDA levels) and a decrease in antioxidant defense markers (GSH content and SOD activity), thereby providing a reliable benchmark for evaluating the comparable redox-disruptive effects of LSP and LSP2. All measurements were performed in triplicate to ensure reproducibility, and absorbance readings were obtained using a microplate reader with appropriate wavelength settings for each assay.

### 4.12. Detection of Apoptosis and Cell Cycle

Seed A549 cells at a density of 1 × 10^5^ cells per well in a 6-well culture plate and culture for 72 h, before exposure to LSPS2 (50, 100, or 200 μg mL^−1^) or LSP (100 μg mL^−1^) for an additional 72 h. The effect of LSP on cell death was detected using a Hoechst staining kit (Beyotime Institute of Biotechnology, Shanghai, China). After two PBS washes, cells were incubated with Hoechst 33342 (5 μg/mL) at 4 °C in the dark for 30 min. Subsequently, Leica DM IRB (Wetzlar, Germany) was utilized to capture fluorescence images of the cells.

### 4.13. Statistical Analysis

Data are expressed as the means ± SD. Two-group comparisons were performed with Student’s *t*-test, and multi-group comparisons with one-way ANOVA followed by Tukey’s post hoc test (GraphPad Prism 9.5). *p* < 0.05 was regarded as statistically significant.

## 5. Conclusions

LSPS2, isolated and purified from *L. sulphureus* via an ultrasound-assisted enzymatic method followed by Fehling’s reagent purification, was structurally characterized as a heteropolysaccharide largely composed of Glc with minor Man and Gal. Structural analyses revealed a backbone of (1→3)-β-D-Glc*p* and (1→4)-α-D-Glcp residues, branched at O-6 and O-3 positions. Both LSP and LSPS2 inhibited A549 cell viability time- and dose-dependently, with LSPS2 showing stronger activity. Mechanistically, LSPS2 induced oxidative stress via increased MDA content, depletion of reduced GSH, and inhibition of SOD activity, leading to apoptosis. While further studies across diverse cancer models are essential, these results suggest LSPS2 warrants further investigation as a potential candidate for the development of anti-cancer agents or functional food candidates targeting oxidative stress pathways.

## Figures and Tables

**Table 1:** Linkages analysis of LSPS2 using methylation analysis.

Time (min)	Partially Methylated Alditol Acetates	Linkages	Molar (%)
16.155	1,5-Di-O-acetyl-1-hydrogen-2,3,4,6-tetra-O-methyl-D-glucitol	T-Glc*p*	18.8
16.295	1,5-Di-O-acetyl-1-hydrogen-2,3,4,6-tetra-O-methyl-D-mannitol	T-Man*p*	1.4
21.800	1,3,5-Tri-O-acetyl-1-hydrogen-2,4,6-tri-O-methyl-D-glucitol	1,3-Glc*p*	42.4
22.381	1,4,5-Tri-O-acetyl-1-hydrogen-2,3,6-tri-O-methyl-D-glucitol	1,4-Glc*p*	18.9
22.988	1,5,6-Tri-O-acetyl-1-hydrogen-2,3,4-tri-O-methyl-D-mannitol	1,6-Man*p*	1.3
30.096	1,3,4,5-Tetra-O-acetyl-1-hydrogen-2,6-di-O-methyl-D-glucitol	1,3,4-Glc*p*	3.2
30.397	1,3,5,6-Tetra-O-acetyl-1-hydrogen-2,4-di-O-methyl-D-glucitol	1,3,6-Glc*p*	14.1

**Table 2:** Chemical shift assignments of ^13^C and ^1^H signals for LSPS2.

Residues	Linkage Type	C1	C2	C3	C4	C5	C6	
		H1	H2	H3	H4	H5	H6a	H6b
A	β-D-Glc*p*-(1→	102.93	73.02	-	69.42	-	60.65	
		4.56	3.35	-	3.46	-	3.93	3.78
B	3→)-β-D-Glc*p*-(1→	102.48	73.02	84.04	68.07	75.49	60.65	
		4.83	3.61	3.83	3.55	3.54	3.93	3.78
C	4→)-α-D-Glc*p*-(1→	100.01	71.45	73.24	76.84	72.79	60.43	
		5.39	3.61	4.00	3.68	3.78	3.90	4.12
D	3,4→)-α-D-Glc*p*-(1→	98.66	-	79.99	-	-	60.43	
		5.02	-	3.94	-	-	3.90	4.12
E	3,6→)-β-D-Glc*p*-(1→	102.70	71.22	84.26	-	-	66.72	
		4.56	3.89	3.81	-	-	4.19	3.72

## Data Availability

The original contributions presented in the study are included in the article/Supplementary Materials, further inquiries can be directed at the corresponding author.

## Supplementary Materials

The following supporting information can be downloaded at: https://www.mdpi.com/article/10.3390/molecules30183706/s1, Figure S1. Methylation analysis of *L. sulphureus* polysaccharide (LSPS2) as determined by gas chromatography–mass (GC-MS). (a–g) The ion fragments of partially methylated alditol acetates (LSPS2s).
