# Supplementary material for: Structural Characterization and Anti-Tumor Activity of a Polysaccharide from Laetiporus sulphureus in A549 Cells
Source: Molecules. 2025 Sep 11;30(18):3706. doi: 10.3390/molecules30183706 (PMC12472605; doi:10.3390/molecules30183706)
Supplement: Supplementary file 1 [file molecules-30-03706-s001.zip › molecules-3832519-supplementary.pdf]

Supplementary materials for

Structural characterization and anti-tumor activity of a poly-saccharide from *Laetiporus sulphureus* in A549 Cells

Yunhe Qu[1], Xing Yang[1,2], Dongxue Zhao[2], Pingping Zhang[1,2], Yue Mi[1,2], Jing Xu[1,2], Boya Zhao[1,2], Dongfang Shi[1]*

[1] Central Laboratory, Changchun Normal University, No. 677 North Changji Road, Changchun, 130032, China

[2] School of Life Science, Changchun Normal University, No. 677 North Changji Road, Changchun, 130032, China

* Correspondence: shidongfang@ccsfu.edu.cn; Tel.: +86-431-86168378

Supplementary figures

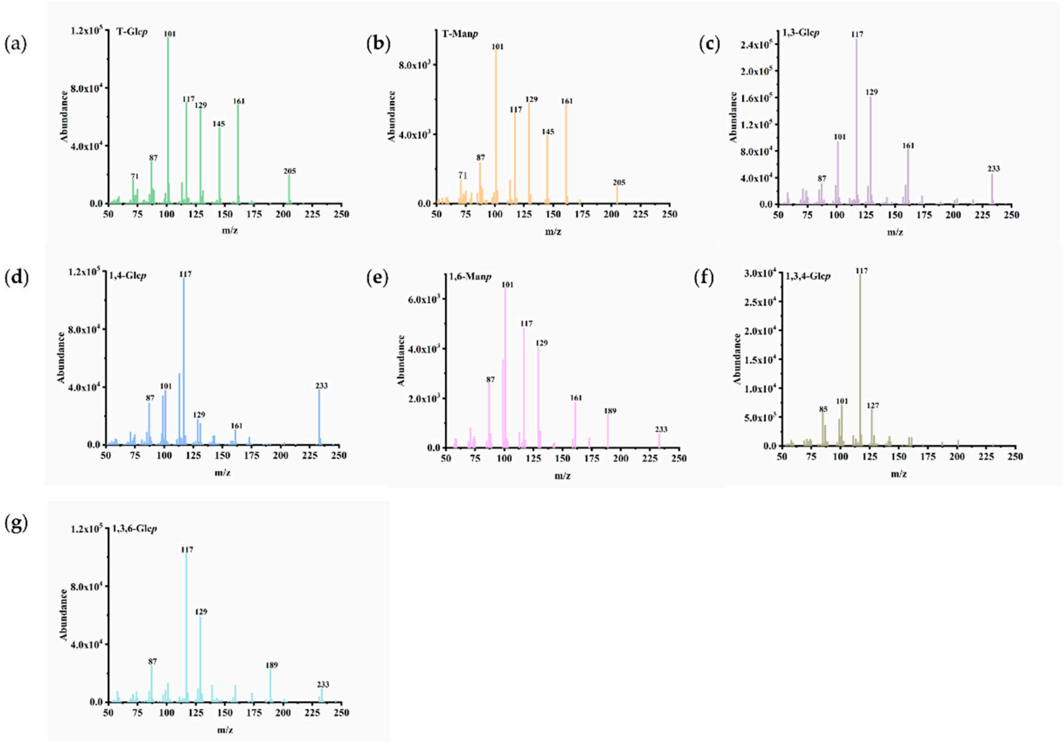

Figure S1. Methylation analysis of *L. sulphureus* polysaccharide (LSPS2) as determined by gas chromatography–mass (GC-MS). **(a-g)** The ion fragments of partially methylated alditol acetates (LSPS2s).
